# Senescence-Associated β-Galactosidase Detection in Pathology

**DOI:** 10.3390/diagnostics12102309

**Published:** 2022-09-25

**Authors:** Yana Valieva, Elena Ivanova, Alexey Fayzullin, Alexander Kurkov, Alexandra Igrunkova

**Affiliations:** 1 Department of Experimental Morphology and Biobanking, Institute for Regenerative Medicine, Sechenov First Moscow State Medical University (Sechenov University), 119991 Moscow, Russia; 2 Department of Pathology, B.V. Petrovsky Russian Research Centre of Surgery, 119991 Moscow, Russia; 3 World-Class Research Center “Digital Biodesign and Personalized Healthcare”, Sechenov First Moscow State Medical University (Sechenov University), 119991 Moscow, Russia

**Keywords:** cellular senescence, aging, senescence-associated beta-galactosidase, replicative senescence, bioimaging, targeted therapy, pathology

## Abstract

Activity of β-galactosidase at pH 6 is a classic maker of senescence in cellular biology. Cellular senescence, a state of highly stable cell cycle arrest, is often compared to apoptosis as an intrinsic tumor suppression mechanism. It is also thought that SA-β-gal is crucial in malignant cell transformation. High levels of senescence-associated β-galactosidase (SA-β-gal) can be found in cancer and benign lesions of various localizations making the enzyme a highly promising diagnostic marker for visualization of tumor margins and metastases. These findings facilitate the research of therapy induced senescence as a promising therapeutic strategy. In this review, we address the need to collect and analyze the bulk of clinical and biological data on SA-β-gal mechanisms of action to support wider implementation of this enzyme in medical diagnostics. The review will be of interest to pathologists, biologists, and biotechnologists investigating cellular senescence for purposes of regenerative medicine and oncology.

## 1. Introduction

β-galactosidase is a lysosomal hydrolase, which cleaves terminal β-d-galactose residues [1]. Substrates of this reaction are β-d-galactosides, such as lactose, keratin sulfates, sphingolipids, etc. [2]. Highly concentrated enzyme can cause disintegration of macromolecular proteoglycans and damage basement membranes through hydrolyzation of glycosidic bonds, resulting in separation of side chains of amino polysaccharides from core proteins [3]. Deficiency of β-galactosidase can lead to galactosialidosis, or Morquio B syndrome. Lysosomal proteases regulate transition of 86-kDa precursor form of β-galactosidase to a 64-kDa mature enzyme. The cathepsin protein is exclusively involved in the maturation of β-galactosidase, while the non-cysteine protease is responsible for the further degradation and inactivation of the enzyme molecule [4].

The activity of β-galactosidase was first discovered by Judith Campisi at pH 4 in dermal fibroblasts and epidermal keratinocytes in 1995. She also revealed that senescent cells contain β-galactosidase, which can be detected at pH 6 [5]. Normal somatic cells reach an irreversible arrest of growth and change their functional state after a finite number of divisions. This process is called replicative aging (senescence), and it is a mechanism for suppressing tumor growth and the main cause of general aging of the body.

**Figure 1:**
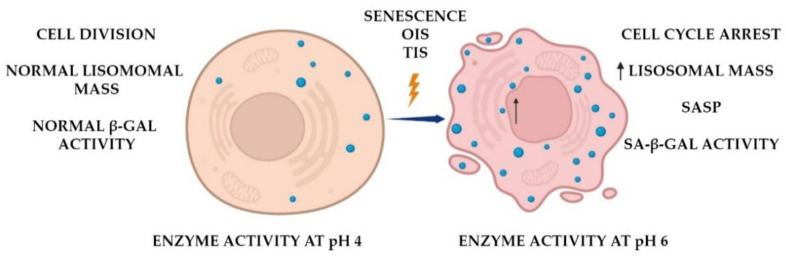
Biological role of senescence-associated β-galactosidase. The enzyme is active in the majority of cells at pH 4, indicating their normal state. Senescent cells accumulate pH 6 active β-galactosidase, undergo cell cycle arrest, and obtain senescence-associated secretory phenotype (SASP) overexpressing GLB1 gene (arrow).

To date, it is known that most mammalian cells, regardless of their age, express lysosomal β-galactosidase activity at pH 4. The enzyme shows its maximum activity at pH 4, but it only can be used to distinguish between “old” and “young” cells using certain biochemical reactions at pH 6 [2]. Detection of β-galactosidase at higher pH became the standard for identification of senescent cells, and the marker referenced as senescence-associated β-galactosidase (SA-β-gal) (Figure 1) [6].

In 2000, it was identified that replicative age correlates with the content of lysosomes and β-galactosidase. The authors suggested that SA-β-gal accumulation indicates residual lysosomal activity at a suboptimal pH in senescent cells [7]. In 2003, it was demonstrated that the presence of autophagic vacuoles correlated with elevated β-galactosidase activity in senescent cells [8]. In 2006, Lee et al. confirmed the lysosomal origin of SA-β-gal activity, showing that it is the result of overexpression of GLB1, the gene encoding the classic lysosomal enzyme [6]. The GLB1 gene is located on chromosome 3p21.33 and encodes a protein of 677 amino acids [9].

The SA-β-gal assay is very useful for testing whether different conditions or compounds can induce or inhibit the appearance of senescent cells. However, a limitation of the assay is that SA-β-gal activity is not a completely specific marker of cellular senescence [10]. The activity of β-galactosidase can be investigated by several methods. The simplest and most commonly used method is cytochemical analysis. Cytochemical analysis is based on staining of cells containing an active enzyme with X-gal chromogen (5-bromo-4-chloro-3-indoyl β-d-galactopyranoside). This chromogen is a direct substrate for β-galactosidase. β-galactosidase cleaves the substrate into galactose and 5-bromo-4-chloro-3-hydroxyindole. The latter product is oxidized to a blue substance providing visual analysis of β-galactosidase activity [11]. Bioluminescence imaging techniques can be applied to detect the enzyme (i.e., flow cytometry [7] or ratiometric probes for fluorescence imaging [12]).

The SA-β-gal assay is irreplaceable when studying how different conditions can impact cellular senescence. It also makes it possible to investigate the potency of drugs and genetic manipulations to induce the aging process in cell cultures or in vivo (tissue samples). Finally, SA-β-gal can be applied to detect and analyze the appearance of senescent cells subjected to stress factors and to identify potential anti-aging protective effects of the compounds [10].

## 2. Medical Applications of SA-β-Gal Assay

SA-β-gal assay found its place in the routine work of thousands of biological laboratories. There is a bulk of evidence that supports the future implementation of β-galactosidase detection as a prognostic marker in medical practice as well [13]. The expression of this enzyme in cells increases in many pathological conditions, especially in inflammation, various dystrophies, and tumors [14].

Oncopathology is the area in which studies of β-galactosidase activity are most numerous [15]. This enzyme can be detected both in benign formations and precancerous conditions and in malignant tumors. It is known that defects in proteins that regulate the cell cycle are common triggers causing the development of cancer. Rearrangement, amplification, and overexpression of cyclins D1, E, and A occur both in hematopoietic diseases and carcinomas of various organs. β-galactosidase can be detected in neoplasias of various organs, which puts it on par with such universal cell cycle-associated proteins as p53, bcl2, PTEN, ki-67. The enzyme is considered to be a product of a suppressor gene that provides prevention of tumor cell transformation [16]. Evaluation of SA-β-gal expression will give a significant impetus to the development of diagnostics of many tumors of various localizations and understanding of their morphogenesis, as well as help in determining the tactics of patient management.

### 2.1. Mechanism of β-Galactosidase Activation

**Figure 2:**
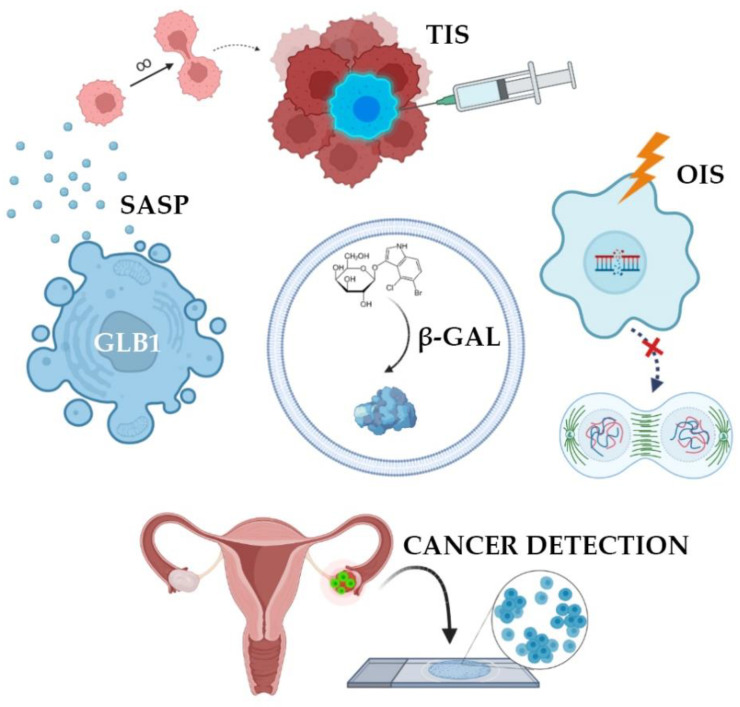
Clinical applications of SA-β-gal detection. SA-β-gal activity is the result of overexpression of GLB1 gene. Normally, oncogene-induced senescence (OIS) prevents cells from becoming tumors. This state can be artificially summoned in order to stop cancer growth (therapy-induced senescence, TIS). Cells accumulate SA-β-gal and acquire senescence-associated secretory phenotype. Such cells do not proliferate but produce and extract signaling molecules forcing surrounding cells into malignant transformation. Finally, some tumors and precancerous lesions have high levels of SA-β-gal, making it a potential prognostic marker and a detection probe.

In many studies, β-galactosidase is perceived as a tumor marker, the increased activity of which directly correlates with the presence of malignant potential in cells [16]. Oncogene activation is known to cause oncogene-induced senescence (OIS) [17]. OIS arrests the cell cycle and limits the proliferative potential of tumor cells [18]. Initially, the mechanism of senescence was studied in detail and described for somatic non-tumor cells, however, then it was later found in atypical cells. OIS results in DNA damage, which activates the p53-p21-p16 gene cascade [19]. This multi-step process overexpresses SA-β-gal [14]. As a result, this innate anticancer mechanism stops the cell cycle of atypical cells. Such cells are no longer able to enter mitosis, therefore, tumor growth stops due to the accumulation of extensive and irreversible damage to telomeric and/or non-telomeric DNA (Figure 2) [20].

### 2.2. Senescence-Associated Secretory Phenotype

Senescence may contribute to the development of cancer by altering the cellular microenvironment through the acquisition of senescence-associated secretory phenotype, SASP [18]. In this state, the cells themselves do not divide but secrete many biologically active substances, such as growth factors, chemokines, cytokines, proteases, and SA-β-gal. These substances are able to paracrinely influence neighboring cells, promoting their active proliferation [14]. SA-β-gal destroys vascular basement membranes and affects the peri-tumor environment [3], promoting tumor growth, invasion, and metastasis [21]. However, atypical cells, while expressing features of immortality, may also accumulate markers of senescence in response to treatment. SASP has been identified in tumors exposed to radiation and chemotherapeutic agents such as cisplatin, carboplatin, doxorubicin, and etoposide [22]. In theory, the negative impact of SASP components on the body can be weakened by removing senescent cells. It is worth noting that senescent cells may be subjected to mutational effects and reactivate their proliferative activity, which will lead to the emergence of various tumors. All in all, senescent mechanisms protect cells from transformation in early age, while prolonged senescence often promotes cancer [18].

### 2.3. β-Galactosidase Dependent Therapy

A major trend in oncology revolves around strategies bringing cancer cells into senescence and then triggering their selective death using senolytic therapy [13]. There are many senolytic drugs that kill senescent cells and are used as chemotherapy drugs. This therapeutic approach is called therapy-induced senescence (TIS) [22]. Senolytics selectively initiate the death of senescent cells responsible for a toxic environment around them, leading to a decrease in the number of secreted chemokines, inhibition of inflammation, and the possibility of restoring the microenvironment by neighboring cells that do not have breakdowns in the genome. To prevent further proliferation under the influence of SASP, it is necessary to eliminate these cancerous senescent cells with special senolytic agents [13]. It should be noted that senolytics are not exclusive to oncology but are also used to treat many inflammatory processes: Arthritis, dementia, heart failure. It has been proven that the activity of SA-β-gal increases in lysosomes of senescent cells, which is a target for the senolytic drug SSK1. This substance was developed on the basis of gemcitabine, a cytotoxic drug from the group of pyrimidine antagonists [23]. SSK1 is specifically activated by SA-β-gal and induces the elimination of “aged” cells. However, it has been suggested that the clinical progression of the tumor during treatment with senolytic drugs may be associated with the ability of atypical cells to avoid undergoing senescence. To solve this problem, it is proposed to combine senolytics with chemotherapy or radiation therapy.

### 2.4. SA-β-Gal Analysis in Oncopathology

The presence of senescent atypical cells has also been found in cultures of primary breast, colon, and prostate cancer cells, as well as in breast cancer, lung cancer, and melanoma cells. These findings stimulated the search for clinically valuable biomarkers of senescence in patient tissues, in particular, SA-β-gal (Table 1).

As the literature data accumulated, it became obvious that the role of SA-β-gal in oncogenesis is not so simple and straightforward. Many studies indicate that it is the cells of primary tumors that contain active SA-β-gal. The activity of the studied enzyme was found in 100% of cases of primary ovarian cancer before patients underwent chemotherapy [24]. SA-β-gal levels were also measured in homogenates of ovarian tumors. In tumor tissues, the content of SA-β-gal is 50% higher compared to the content of this enzyme in healthy tissue [25]. The expression of other cellular markers of senescence was also quite significant in primary epithelial ovarian tumors, particularly phosphorylated form of histone family member X (γ-H2A.X) and tumor suppressor p53 binding protein (53BP1) [23].

**Figure 3:**
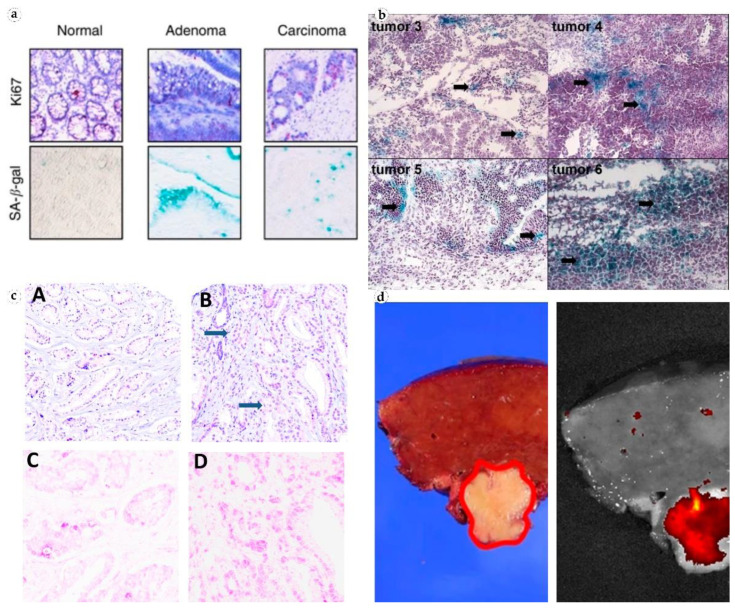
Visualization of SA-β-gal in patient tissues. (**a**) Non-senescent normal colonic crypt mucosa, senescent colon adenoma, and a formally non-senescent colon carcinoma with numerous senescent cells. Reproduced from [27] under the terms of the Creative Commons CC BY license; (**b**) senescent cancer cells in ovarian tumors from patients who did not receive chemotherapy. Reproduced from [24] under the terms of the Creative Commons CC BY-NC-SA license; (**c**) prostate cancer tissue, H&E (**top**), and antibodies to GLB1 (**bottom**). Reproduced from [33] under the terms of the Creative Commons CC BY license; (**d**) fluorescence imaging of β-galactosidase in freshly resected human hepatocellular carcinoma. Reproduced from [36] under the terms of the Creative Commons CC BY license.

In colorectal cancer cells, SA-β-gal is expressed both before and after chemotherapy. In malignant tumors of the large intestine, the level of the enzyme was two times higher than in normal tissue [26]. Colon cancer biopsies comprised “senescent sites” after treatment with 5-fluorouracil and leucvorin. The presence of such sites correlated with a higher survival rate among patients (Figure 3) [27]. In addition, serum SA-β-gal activity has been found to be elevated in invasive colon tumors [46]. SA-β-gal is also active in precancerous colon adenomas [28]. Studies in colorectal cancer have shown that TIS of tumor cells contributes to the overall outcome of chemotherapy with DNA-targeting drugs [26].

### 2.5. SA-β-Gal Detection

New imaging techniques make it possible to detect tumor cells with high SA-β-gal content in situ. Ratiometric probes are currently being actively developed for bioluminescent imaging for cancer detecting in vivo. Activatable fluorescent probes are designed to become fluorescent only after they come in contact with the target tissue and are activated by SA-β-gal [47]. Another ratiometric probe allows visualizing SA-β-gal in colorectal cancer in real time in vivo during fluorescence endoscopy. In vivo real-time capture of β-galactose activity was performed at tumor site with a high-resolution three-dimensional view [12]. Two-photon fluorescent probe FC-β-gal was designed to visualize endogenous β-galactosidase in lysosomes in ovarian cancer cells, presenting an original approach to visualization and diagnosis of primary ovarian cancer cells [48]. Photometric imaging therapy with a macrotheranostic probe was tested to visualize cancer cells. Overexpressing SA-β-gal cells were identified through near-infrared fluorescence, photoacoustic and photothermal signals presenting new possibilities for imaging-guided cancer therapy [49]. It is also possible to detect SA-β-gal in metastatic cells by bioluminescent imaging. This approach was implemented for metastases of ovarian [50] and gastric [42] cancers.

Surprisingly, when studying SA-β-gal activity in gliomas, it was found that the activity of the enzyme was significantly higher in solid than in infiltrating tumors. The greatest increase in SA-β-gal activity was observed in oligodendroglioma, but all solid glial tumors expressed more SA-β-gal than infiltrating tumors [21]. These findings indicate that accumulation of the enzyme in tumor tissues may play a role in an innate anti-metastasis mechanism.

### 2.6. SA-β-Gal as a Prognostic Marker

SA-β-gal is active not only in tumor cells but also in cells of precancerous lesions making this enzyme a potentially valuable prognostic marker. Activity of SA-β-gal was studied in a series of breast tumors: Fibroadenoma (non-invasive benign mesenchymal tumor), proliferative fibrocystic mastopathy (which is a precancerous condition), and infiltrative breast carcinoma. The study revealed that SA-β-gal activity was normal in fibroadenoma cells, increased in cells of proliferating ducts in fibrocystic mastopathy, and at maximum level in infiltrative breast carcinoma cells [26]. These results showed a strong correlation between the levels of SA-β-gal and the malignant potential of cells.

Immunohistochemical expression of β-galactosidase in prostatic intraepithelial neoplasia of high severity (PIN III) and in glandular-stromal hyperplasia without atypia was significantly elevated. However, the level of this enzyme was lower in primary prostate cancer tissues, and minimum detection was observed in the tissue of a healthy prostate. This could be attributed to the increase in β-galactosidase activity during the early period of cell senescence which could also decrease with time. The authors also suggest that the presence of a large number of senescent cells in more highly differentiated tumors may be associated with their indolent course [31]. The activity of SA-β-gal correlated with the severity of prostatic hyperplasia in glandular-stromal hyperplasia. Prostate epithelial cells expressed SA-β-gal in patients with more pronounced prostate enlargement (mass > 55 g), while senescent cells were absent in prostates weighing <55 g [32].

SA-β-gal is also expressed in 60% hepatocellular carcinoma tissues. In normal liver tissue, SA-β-gal activity is detected in 20% of cases, while it was found in 50% of patients with fibrosis of chronic viral hepatitis C ethiology [34]. The authors suggest that this is due to the fact that chronic hepatitis facilitates accelerated replicative aging of the cells, accumulating senescent cells and predisposing them to the development of hepatocellular carcinoma.

SA-β-gal can be considered a marker of tumor response to treatment useful for assessing the prognosis of the disease. It was previously thought that the use of cytotoxic drugs in cancer therapy results in disease stabilization rather than tumor regression, and this was generally regarded as a treatment failure. However, it has been shown that DNA damage can induce senescence in tumor cells expressing wild-type p53. Cytotoxic drugs are able to induce tumor tissue aging in vivo by activating the p53, p21, and p16 cascade and overexpressing SA-β-gal, which supports the aging process in cells. Not unlike apoptosis, senescence appears to be a p53-induced cellular response to DNA damage and an important factor in determining treatment outcomes [34]. For most somatic cancers, induction chemotherapy without concomitant radiation therapy provides 20% to 40% response to the disease, with >95% of these responses being a partial reduction in tumor volume, which in theory may be associated with activation of senescence and SA-β-gal [51,52].

### 2.7. Chemotherapy and Cellular Senescence

In breast cancer, 41% of tumors contain cells with active SA-β-gal after a course of neoadjuvant therapy. It is much higher than 2% frequency of SA-β-gal positive tumors among patients who did not undergo chemotherapy [30].

In prostate cancer tissues, β-galactosidase expression increases after neoadjuvant therapy, mainly among more clinically favorable types of moderate-severity cancers. In addition, androgen withdrawal has also been noted to induce cellular senescence in prostate tumors [33]. Given concerns about the long-term persistence of SASP cells, targeting the removal of these cells from the body may improve clinical outcomes.

The effect of chemotherapy on cellular senescence was demonstrated by studying SA-β-gal activity in lung cancer cells before and after therapy. The enzyme was not expressed in samples from patients who did not receive chemotherapy. The results were positive only in tumors excised after neoadjuvant therapy. Tumors that exhibited active SA-β-gal had reduced initial volume and retarded growth after chemotherapy [37].

### 2.8. SA-β-Gal in Non-Tumor Lesions

SA-β-gal is expressed in both malignant and benign neoplasms (i.e., in melanocytic nevi). Results of studies on SA-β-gal activity in nevi are contradicting. In the study of neonatal nevi, the enzyme was expressed in 100% of cases [38]. However, another research group proved through a rigorous methodology that adult melanocytic nevi do not contain any SA-β-gal with activity at pH 6 [39]. This difference could be attributed to differences in patient’s age or protocols which was brought up in the latter study.

There are other non-tumor pathological conditions in which active SA-β-gal is detected in tissue cells. The enzyme content in chondrocytes of articular cartilage correlates with osteoarthritis severity [40]. Moreover, SA-β-gal activity is increased in endotheliocytes of vessels affected by atherosclerosis [14].

It has been suggested that the immune response to inflammation may depend on the senescence of peripheral blood cells. It was shown that the level of SA-β-gal increased depending on the age of the donor during the cultivation of his peripheral blood mononuclear cells. The greatest age-related increase was observed in populations of CD8+ T cells, in which the proportion of cells with high SA-β-gal activity reached 64% in 60-year-old donors. CD8+ T cells with high levels of SA-β-gal had shortened telomeres and upregulated p16 pathway. This led to a disruption of proliferation and differentiation in T cells [53].

### 2.9. SA-β-Gal in Fibrosis

A number of studies report correlations between SA-β-gal expression in fibroblasts and the severity of fibrotic processes in connective tissue. Similar to tumors, senescent fibroblasts gradually increase p53, p21WAF1, and p16INK4a levels, produce high levels of reactive oxygen species, and accumulate oxidative DNA damage [54,55]. Gradually this leads to overproduction of collagen and fibrotic transformation of the tissue. It is also possible that the ability of serum growth factors to modulate senescent cells can be connected with the upregulation of collagenase synthesis. After such stimulation, the culture of “young” fibroblasts responded with an increase in the levels of procollagenase and tissue inhibitor of metalloproteinases. However, senescent fibroblasts produced relatively high levels of procollagenase in low serum concentrations, did not increase procollagenase synthesis, and expressed TIMP at low levels [56]. Other authors showed that β-galactosidase was present in senescent fibroblasts and keratinocytes but was absent in resting fibroblasts and terminally differentiated keratinocytes. An increase in this marker in fibroblasts and epidermal keratinocytes depended on the age of the donor [5]. When studied in vitro, fibroblasts excised from elderly people had higher SA-β-gal activity compared to fibroblasts from younger people. Similar results were obtained when assessing age dependence of cell apoptosis [57].

Researchers from China investigated the effect of cigarette smoke extract on the growth, proliferation, and aging of skin fibroblasts and the possible mechanism underlying these effects. Fibroblasts exposed to even low concentrations of cigarette smoke for a long period of time (5 passages) showed significantly increased SA-β-gal activity and typical signs of “aging” cells [58].

However, there are studies that show that cellular aging does not always lead to an increase in SA-β-gal activity. Skin isolated fibroblasts with defective lysosomal β-galactosidase from patients with autosomal recessive GM1 gangliosidosis did not express SA-β-gal at late passages, even though they underwent senescence. In addition, normal late-passage fibroblasts with interfering RNA depleting GLB1 mRNA underwent senescence, but SA-β-gal expression was not found in them. GLB1 mRNA depletion also prevented expression of SA-β-gal activity in HeLa cervical carcinoma cells that had undergone senescence by suppressing their endogenous human papillomavirus E7 oncogene. SA-β-gal induction during senescence is probably driven by increased expression of the lysosomal protein β-galactosidase [6]. The authors suggest that lysosomal β-galactosidase is the source of SA-β-gal activity in senescent cells and indicate that SA-β-gal is not a specific marker of aging per se, but rather a surrogate marker for an increase in the number or activity of lysosomes, which have long been associated with replicative aging and general body aging.

Senescent fibroblasts were found in lung tissues of patients with idiopathic pulmonary fibrosis. These cells expressed high levels of β-galactosidase, p21, p16, p53, and SASP-associated cytokines wile proliferation and apoptosis were inhibited. When treated with transforming growth factor-β, senescent pulmonary fibroblasts obtained shortening of telomeres, mitochondrial dysfunction, and elevated markers of endoplasmic reticulum stress [59]. It is known that upregulated miR-34a is strongly associated with senescence of fibroblasts in lungs and reduces the proliferation of cells [60].

## 3. Future Prospects

SA-β-gal is a very promising target for both diagnostics and therapy. It became a widely used biomarker of cellular senescence since it is very easy to detect and study both in vivo and in vitro. It should be noted that this enzyme is associated not only with cell cycle arrest but also with many other pathological processes, especially tumors. The unique characteristics of this enzyme, when further studied, may show its importance as a tumor suppressor. However, many enzyme detection reports contradict each other leaving the exact tissue- and condition-specific functions of SA-β-gal debatable.

In the present review, we attempted to summarize all information on pathology perspective on SA-β-gal to date. The major advantage of SA-β-gal detection in comparison to other markers of senescence is its innate enzymatic activity making it a target for theranostics. The relative simplicity of SA-β-gal visualization protocols requiring only chromogen with proper buffer solutions and oxidants (i.e., nitro blue tetrazolium) creates a possibility for highly reproducible and accurate detection tests. Direct staining of non-processed tissues allows immediate in situ diagnostics which corresponds to the trends in digital pathology [12,47,48]. However, while it is clear that SA-β-gal is a useful tool for tissue analysis, the actual value of its detection remains debatable. After accumulating the information from research papers presenting the results of the biochemical reactions, we highlighted the following major aspects of SA-β-gal detection that can be of value in oncological diagnostics:

(1) SA-β-gal accumulation in tumors is non-linear, which can be used to evaluate the progression of precancerous and cancerous lesions. Some studies reported that the expression of SA-β-gal strongly correlated with an increase in the malignant potential of a cell: The highest level of SA-β-gal was detected in carcinomas, the average level was in precancerous conditions and the lowest level was in benign tumors [26,31,45]. However, it is likely that high expression of β-galactosidase is temporary and reaches its peak in obligate precancers or in recent carcinomas [31,43]. Prolonged clinical studies are needed to shed light on the dynamics and possible impact of cellular senescence on tumor growth.
(2) SA-β-gal expression differs among cancer cells. It can be explained by the heterogenous structure of tumors and functional distinctions between primary tumor cells and metastases. Although SA-β-gal was used to visualize ovarian and gastric cancer metastases by bioluminescence imaging [42,50], the enzyme reached its highest concentration in solid non-invasive tumors [21]. This can be used to better evaluate the risk of tumor progression, i.e., after surgical resection.
(3) SA-β-gal content significantly increases after neoadjuvant therapy [30,37]. This can be used to assess patient’s response to the treatment and correct therapeutic strategy. Targeting SA-β-gal for the delivery of senolytic drugs has a chance to become a mainstream direction in the treatment of tumors or inflammatory processes. Cellular senescence may explain the persistence of some tumor cells and may become important in predicting response to therapy. The exploration of SA-β-gal activity in the development of antifibrotic drugs for hepatic fibrosis, hypertrophic scars, and other conditions is also very promising.

## 4. Conclusions

Research into SA-β-gal activity reveals major opportunities in developing diagnostic and therapeutic approaches to inflammatory diseases, precancerous conditions, and cancer. Our review of articles reporting results of biochemical reactions from different fields of medicine revealed that SA-β-gal accumulation in tumors is non-linear, organ-specific, and depends on the duration of disease and its progression. Detection of SA-β-gal has the potential to become an important assay for personalized tumor treatment. Further studies are needed to uncover the relations between cellular senescence and pathological processes.

## Figures and Tables

**Table 1:** Research studies with results of SA-β-gal analysis in patient tissues.

Condition	Number of Patients	SA-β-Gal Localization	Results	Reference
Primary ovarian cancer	11	Cells of ovarian cancer not subject to chemotherapy	SA-β-gal activity was detected in 100% of cases of primary ovarian cancer	[24]
Epithelial ovarian adenocarcinoma	40	Healthy tissues and epithelial ovarian adenocarcinoma	SA-β-gal activity in tumor tissues is 50% higher than in healthy tissues	[25]
Colon adenocarcinoma	7	Colon adenocarcinoma tissue	SA-β-gal activity in adenocarcinoma tissues is 2 times higher than normal	[26]
Colon adenoma and untreated invasive carcinoma	23	Senescent cells within neoplastic epithelial areas of manifest colorectal carcinomas	SA-β-gal activity was found in 8 out of 12 adenomas, 1 of out 6 invasive carcinomas, 0 out of 5 normal crypt mucosa tissues	[27]
Colon adenoma	59	Cells of colon adenomas	A strong correlation between SA-β-gal staining and senescence immunohistochemical markers	[28]
Glioma	23	Cells of malignant glial tumors	The greatest increase in the activity of β-galactosidase was in anaplastic oligodendroglioma; in other glial tumors, it was also higher than in meningiomas and metastatic tumors	[21]
Glioma	5	Tumor-associated brain endothelial cells	SA-β-gal activity was detected in glioma tissue cells, while normal brain tissue was negative	[29]
Breast cancer, fibroadenoma, fibrocystic disease	18	Carcinomas, fibrocystic, fibroadenomatous tissues	SA-β-gal activity was normal in fibroadenoma cells, increased in fibrocystic cells, and maximum level was observed in breast cancer cells	[26]
Breast cancer	56	Tissues of treated and untreated breast tumors	SA-β-gal activity was detected in 15 of 36 tumors. Tumor sections of patients who had not received chemotherapy expressed SA-β-gal in 2 of 20 cases	[30]
Prostate cancer, benign prostatic hyperplasia, high-grade prostatic intraepithelial neoplasia	124	Human prostate epithelial cells	High expression of SA-β-gal was observed in 37% of primary cancer specimens, 72% of high-grade prostatic intraepithelial neoplasia samples, and 13% of benign tissues	[31]
Benign prostatic hyperplasia	43	Human prostate epithelial cells of hypertrophied tissues	SA-β-gal activity was detected in 17 out of 43 specimens. Prostate epithelial cells expressed SA-β-gal in patients with more pronounced prostate enlargement weighing more than 55 g, while the senescent cells were absent in prostate cells weighing less than 55 g	[32]
Prostate cancer	126	Senescent prostate cancer cells	GLB1 staining was expressed at higher levels in prostate samples treated with androgen deprivation	[33]
Chronic hepatitis C, hepatocellular carcinoma	57	Replicating cells in normal liver, liver with chronic hepatitis C and hepatocellular carcinomas	In normal liver tissue, SA-β-gal activity can be detected in 20% of cases. SA-β-gal was expressed in 60% of hepatocellular carcinoma samples and in 50% of samples with hepatitis C	[34]
Chronic viral hepatitis	20	Tissues of liver with chronic viral hepatitis B, chronic viral hepatitis C, and normal liver	The SA-β-gal activity was frequently detected in periportal or periseptal hepatocytes of liver cirrhosis and focally in chronic hepatitis irrespective of type B or type C infection, while the enzyme activity was extremely weak in normal livers	[35]
Hepatocellular carcinoma	95	Cancer cells	High SA-β-gal activity in tumor and low SA-β-gal activity in non-tumor tissues	[36]
Lung Cancer	6	Tumor cells treated with chemotherapy and radiation	The enzyme was not detected in samples from patients who did not receive chemotherapy. SA-β-gal expression elevated in tissues after neoadjuvant chemotherapy	[37]
Melanocytic naevi	23	Tissues of congenital naevi	Human naevi, largely growth-arrested neoplastic lesions, are positive for the senescence marker SA-β-gal	[38]
Melanocytic naevi	17	Adult nevi cells	Every specimen evaluated showed varying degrees of positivity at the optimal pH 4, none of the specimens showed staining at pH 6	[39]
Knee osteoarthritis	50	Chondrocytes in articular cartilage	SA-β-gal staining was found in a subset of chondrocytes close to the lesion site of mild, moderate, and severely altered knee cartilage with osteoarthritis. No SA-β-gal staining was observed in normal articular cartilage samples	[40]
Atherosclerosis	3	Atherosclerotic aorta	The aortic endothelium cells overlying atherosclerotic plaques were SA-β-gal-positive. The endothelium covering nearby regions of relatively normal aorta was SA-β-gal-negative	[41]
Gastric cancer	13	Gastric cancer cells, peritoneum metastatic cells	The SA-β-gal content in the tumor was high. SA-β-gal allowed locating peritoneal metastases	[42]
Intraductal papillary mucinous neoplasm of the pancreas	39	Neoplasms with low-, intermediate- and high-grade dysplasia, associated invasive carcinoma	Senescence is induced in the early stage of intraductal papillary mucinous neoplasm and gradually attenuated according to the progression	[43]
Cervical and endometrial carcinoma	77	Tissues of invasive cervical cancer and endometrial cancer	Squamous cell carcinoma (negative reaction) and cylindrocellular carcinoma (positive reaction in cancer cells)	[44]
Usual type uterine leiomyoma	14 (86 samples)	Fibroid tissue sections	48 out of 82 tumors were SA-β-gal positive in >10% of the tumor volume. The more senescent cells, the higher the stage (1–4) and the lower the growth potential of the tumor	[45]

## Data Availability

Not applicable.
